# Chest Compressions in Pediatric Patients With Continuous-Flow Ventricular Assist Devices: Case Series and Proposed Algorithm

**DOI:** 10.3389/fped.2022.883320

**Published:** 2022-06-21

**Authors:** Ivie D. Esangbedo, Priscilla Yu

**Affiliations:** ^1^Division of Critical Care, Seattle Children's Hospital, University of Washington, Seattle, WA, United States; ^2^Division of Critical Care, University of Texas (UT) Southwestern Medical Center, Children's Medical Center Dallas, Dallas, TX, United States

**Keywords:** pediatric, chest compression (CC), ventricular assist device (VAD), continuous flow ventricular assist device, cardiopulmonary resuscitation (CPR), cardiac intensive care unit, left ventricular assist device (LVAD)

## Abstract

Patients with continuous flow ventricular assist devices (CF-VAD's) in the systemic ventricle (left ventricle or single ventricle) often have no palpable pulses, unreliable pulse oximetry waveforms and non-pulsatile arterial waveforms despite hemodynamic stability. When circulatory decompensation occurs, standard indicators to begin cardiopulmonary resuscitation (CPR) which are used in other pediatric patients (i.e., significant bradycardia or loss of pulse) cannot be applied in the same fashion. In this population, there may already be pulselessness and development of bradycardia in and of itself would not trigger chest compressions. There are no universal guidelines to dictate when to consider chest compressions in this population. As such, there may be a delay in decision-making or in recognizing the need for chest compressions, even in patients hospitalized in intensive care units (ICU) and cared for by experienced staff who perform CPR regularly. We present four examples of pediatric cardiac ICU patients from a single center who underwent CPR between 2018 and 2019. Based on this case series, we propose a decision-making algorithm for chest compressions in pediatric patients with CF-VADs in the systemic ventricle.

## Background

The implantation of ventricular assist devices (VADs) in pediatric patients has become relatively widespread and has increased with time over the past decade (1–3). According to the most recent annual report from the Pediatric Interagency Registry for Mechanical Circulatory Support (Pedimacs), there were over 1,200 device implantations in 1,011 pediatric patients <19 years by North American hospitals between September 2012 and December 2020 (1). The most common devices used were intracorporeal continuous flow devices, accounting for 41% of patients from this time period. An additional 26% were paracorporeal continuous flow devices. Thus, the majority (67%) of pediatric VADs implanted during this time period were continuous flow devices. In considering only the younger non-adolescent pediatric population (<10 years of age), continuous flow device use was still substantial. There were 355 continuous flow VADs implanted (233 paracorporeal, 122 intracorporeal) of 627 device implantations; 56.6% (1).

While Pedimacs and other registries (1–6) collect data on adverse events in pediatric patients with mechanical circulatory support (MCS), there is no published data on the frequency of resuscitation necessitating chest compressions in this population. Though the frequency of discharge home in pediatric patients with VADs has increased over the past decade, a significant number of these patients still have prolonged hospital stays prior to discharge or never get discharged home (5).

Pediatric patients with continuous flow ventricular assist devices (CF-VAD's) in the systemic ventricle (left ventricle or single ventricle) often have no palpable pulses, unreliable pulse oximetry waveforms and non-pulsatile arterial waveforms despite hemodynamic stability. When circulatory decompensation occurs, standard indicators to begin cardiopulmonary resuscitation (CPR) which are used in other pediatric patients (i.e., significant bradycardia or loss of pulse) (7) cannot be applied in the same fashion. In this population, there may already be pulselessness and development of bradycardia in and of itself should not trigger chest compressions. There are no universal guidelines to dictate when to initiate or consider chest compressions in this pediatric population. Further, it is unclear whether existing adult algorithms for CPR in CF-VAD patients (8–10) can be applied in blanket fashion to adolescents, akin to how the American Heart Association's (AHA) algorithm for adult basic life support (BLS) is used in post-pubertal pediatric patients (11). Studies of hospitalized adult CF-VAD patients in cardiovascular emergencies have shown that there is often uncertainty about when to initiate chest compressions leading to delays in resuscitation (8, 9).

We present four cases of pediatric patients with CF-VADs from a single center who underwent in-hospital CPR between 2018 and 2019. Based on this case series, we propose a decision-making algorithm for chest compressions in pediatric patients with CF-VADs in the systemic ventricle.

## Case Series

Table 1 summarizes the four pediatric (<18 years old) patients in this case series. Each patient had a CF-VAD in the systemic ventricle, and each received chest compressions while admitted within the cardiac intensive care unit.

**Table 1:** Summary of patient data.

**Patient**	**Age**	**Diagnosis**	**Type of left VAD**	**Reason for clinician attention**	**Reason chest compressions initiated by clinician**	**Duration of chest compressions**	**CPR outcome**
#1	16 years	Cardiomyopathy, muscular dystrophy	HeartMate-3™ (Abbott^®^, Abbott Park, Illinois)	Telemetry	Asystole	15 min	Neurologic injury, death.
#2	3 years	Pacemaker-induced cardiomyopathy	Jarvik 2015 (JarvikHeart™, New York, New York)	Low-flow alarm	Disconnected RVAD cannula	4 min	Restored MAP and VAD function. No direct sequalae.
#3	17 years	Dilated cardiomyopathy, idiopathic	HeartMate-3™ (Abbott^®^, Abbott Park, Illinois)	Low-flow alarm	Altered mental status; progressive decline of VAD flow until <0.5 L/min	2 min	Restored MAP and VAD function. No direct sequalae.
#4	15 years	Dilated cardiomyopathy, idiopathic	HeartWare™ HVAD™ (Medtronic^®^, Dublin, Ireland)	Low-flow alarm	Altered mental status; progressive decline of VAD flow; MAP <30 mmHg	2 min	Restored MAP and VAD function. No direct sequalae.

*CPR, cardiopulmonary resuscitation; MAP, mean arterial pressure; VAD, ventricular assist device*.

Patient #1 was a 16-year-old, 70 kg (1.8 m^2^) male with muscular dystrophy and dilated cardiomyopathy, who underwent placement of a HeartMate-3™ (Abbott^®^, Abbott Park, Illinois) left ventricular assist device (LVAD) as destination therapy. On post-operative day 10, he developed significantly decreased VAD flows associated with worsening right ventricular function and the development of pericardial tamponade. He was initially planned for an urgent pericardiocentesis. Asystole occurred upon induction of anesthesia, and chest compressions were initiated shortly after, as the VAD flows declined. He instead underwent an emergent subxiphoid pericardial window. Duration of chest compressions was 15 min. There was evidence of significant neurologic injury after the event, which led to withdrawal of life support.

Patient #2 was a 3-year-old, 14 kg (0.56 m^2^) girl with congenital heart block who developed pacemaker-induced cardiomyopathy. As a bridge to heart transplant, she underwent placement of a Jarvik 2015 LVAD (JarvikHeart™, New York, New York) in addition to a PediMag™ (Thoratec^®^, Pleasanton, California) right ventricular assist device (RVAD). On post-operative day 6, while mechanically ventilated, there were low-flow alarms from her RVAD. It was quickly noted that she had had accidental disconnection of her RVAD inflow cannula. This quickly resulted in significant blood loss and hypotension. Her RVAD cannula was quickly reconnected and pump speed resumed. She received 4 min of CPR prior to resumption of full flows. She suffered no neurologic injury and ultimately underwent heart transplantation six months after VAD placement.

Patient #3 was a 17-year-old, 110 kg (2.1 m^2^) male with dilated cardiomyopathy who underwent placement of a HeartMate-3™ LVAD. On post-operative day 9, he was noted to have rising central venous pressures (CVP) and elevated lactate levels, as well as a drop in hemoglobin. He soon developed altered mental status, hypotension, and low-flow alarms from his VAD. Over the course of minutes, he had progressively lower flows. When flows dropped below 0.5 L/min, chest compressions were initiated. He received 2 min of chest compressions, with simultaneous administration of an intravenous fluid bolus and a dose (0.01 mg/kg) of epinephrine. VAD flows were restored, as was mean arterial pressure (MAP). He underwent an emergent mediastinal exploration, which revealed hemopericardium and a hemothorax.

Patient #4 was a 15-year-old, 53 kg (1.5 m^2^) female with dilated cardiomyopathy. She underwent placement of a HeartWare™ HVAD™ (Medtronic^®^, Dublin, Ireland) LVAD. On post-operative day 14, her VAD flows acutely dropped to 0.8 L/min, MAP dropped from 60 to 65 mmHg to <30 mmHg and she became suddenly unresponsive. Prior to this, she had been awake, alert, sitting up, receiving non-invasive mechanical ventilation. Chest compressions were initiated, immediately followed by the administration of an epinephrine dose (0.01 mg/kg) and an intravenous fluid bolus. After a 2-min cycle of CPR, VAD flows increased and MAP normalized. She was subsequently confirmed to have pericardial tamponade, and underwent mediastinal exploration with hematoma evacuation. On post-operative day 24, now sedated and intubated, this patient had another acute drop in VAD flow after administration of a fosphenytoin dose for new-onset seizures. VAD flows again dropped below 1 L/min, and MAP dropped below 30 mmHg. With this second event, mental status changes could not be assessed as she was receiving sedative infusions. She did not receive chest compressions. She was given a dose of epinephrine (0.01 mg/kg) and an intravenous fluid bolus. VAD flows and MAP normalized.

Three of these four patients (#2, #3, and #4) initially came to clinician attention due to low-flow alarms (Table 1). In patient #4, who had two separate but similar low-flow events, the decision to initiate chest compressions only occurred with her first event, and this decision was informed by the acute change in mental status. In patients #1, #3, and #4, there was initial clinician uncertainty about when it was the appropriate time to initiate chest compressions in this population with hemodynamically-stable pulseless electrical activity (PEA) at baseline.

## Decision-Making Algorithm

**Figure 1:**
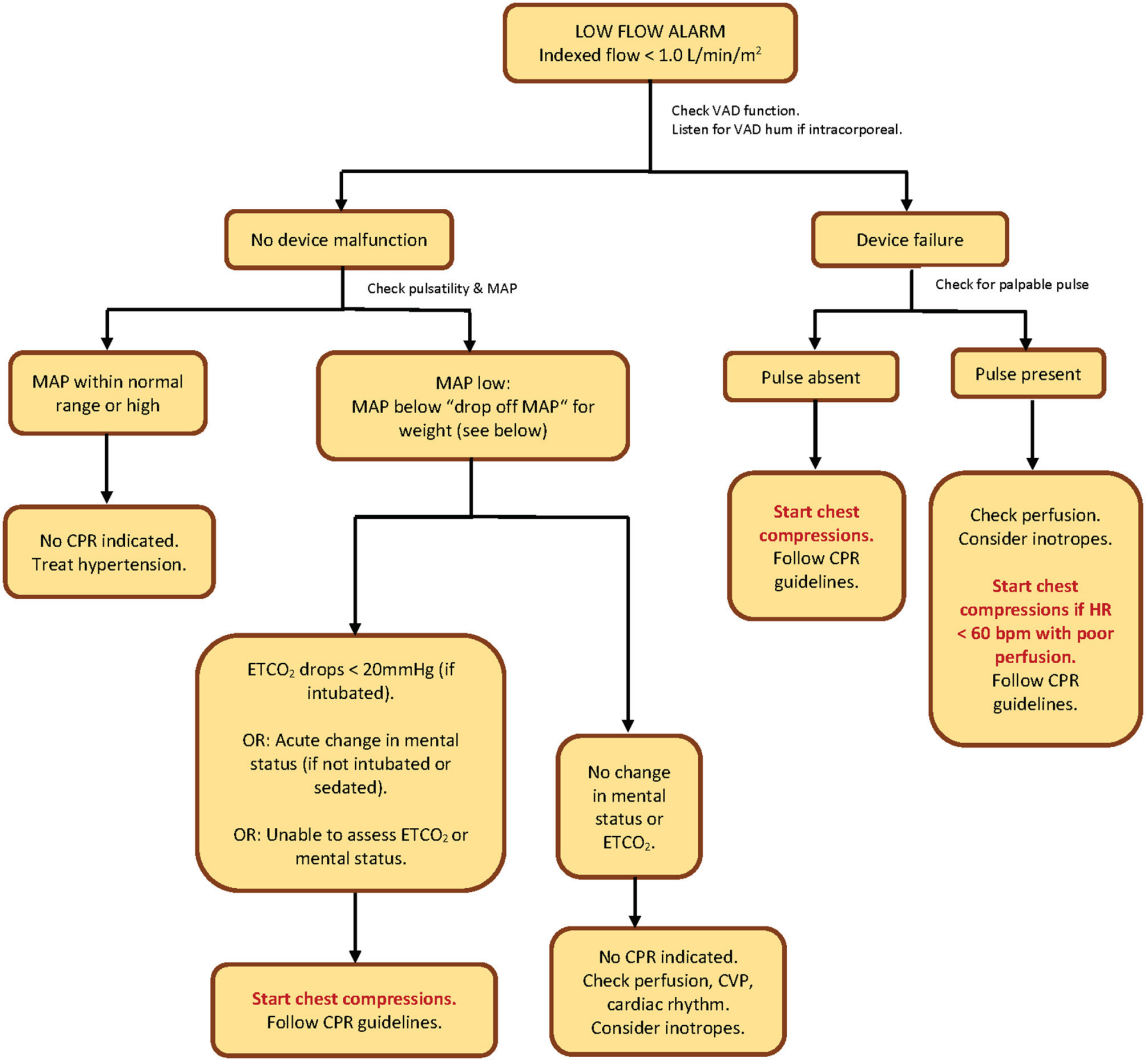
CPR algorithm to determine initiation of chest compressions in pediatric patients with continuous-flow ventricular assist devices in the systemic (left/single) ventricle. “Drop-off MAP” by patient weight: If weight <15 kg = 25 mmHg; if weight 15–30 kg = 30 mmHg; if weight >30 kg = 35 mmHg. VAD, ventricular assist device; MAP, mean arterial pressure; CPR, cardiopulmonary resuscitation; ETCO_2_, end-tidal carbon dioxide; HR, heart rate; bpm, beats per minute; CVP, central venous pressure.

Using the knowledge of sequence of events in these 4 cases, we created an algorithm to guide the decision about initiation of chest compressions in pediatric patients with CF-VADs in the systemic ventricle (Figure 1). We decided to focus simply on an algorithm that informs the decision to begin chest compressions.

Our decision-making algorithm starts with a response to a low-flow alarm. We chose to start here since 3 out of 4 patients in our case series came to clinician attention due to low-flow alarms. Our algorithm is initiated once the indexed VAD flow falls below 1 liter per minute per square meter. We did not start with a patient symptom first (such as change in mental status or hypotension), because in all of our 4 cases, the low-flow alarm preceded every symptom in drawing attention to the patient.

We considered complete VAD failure and the absence of a palpable pulse to be a clear indication for chest compressions. In cases where there is VAD dysfunction but the presence of a pulse, we recommend that the decision to initiate chest compressions be based on poor perfusion associated with a heart rate <60 beats per minute, in alliance with the American Heart Association (AHA) pediatric basic life support guidelines (11).

In patients with low flows with no evidence of VAD dysfunction, the next decision-making steps should be confirmation of non-pulsatile flow (no palpable pulse), followed by determination of mean arterial blood pressure (MAP). MAP measurement can be accomplished invasively *via* intra-arterial catheter or non-invasively *via* the Doppler opening pressure (DOP). Confirmation of non-pulsatile flow prior to MAP measurement by cuff is important since the DOP may over-estimate the MAP and may more closely correlate with systolic pressure if the patient is pulsatile (12). The steps in the algorithm can still be followed if the patient has a narrow pulse pressure, and some studies have demonstrated that DOP is an accurate assessment of MAP even in pulsatile LVAD patients (13).

Excessively high MAP's should be treated to restore flows. For low MAP's, we set cut-off points by weight, which we call the “drop-off MAP.” In those low-flow patients whose MAP's fall below the “drop-off MAP” cutoff, we propose that the decision point for initiation of chest compressions should be acute change in mental status (for non-intubated non-sedated patients) or a drop in invasive end-tidal carbon dioxide (ETCO_2_) to < 20 mmHg (for intubated patients).

Supplementary Table S1 summarizes some patient- and device-related problems to consider in patients with CF-VAD in the left ventricle who develop low-flow or evidence of decreased tissue perfusion.

## Discussion

To our knowledge, no pediatric algorithms exist in the literature to determine the initiation of chest compressions during cardiovascular emergencies in this unique population. CPR algorithms for adult patients with CF-VAD's have been proposed and published in the literature (8–10). In addition, a 2017 AHA statement provides an algorithm for emergency medical personnel responding to unresponsive VAD patients in the field (14).

Studies have demonstrated that chest compressions may be safe and effective in patients with intracorporeal VADs (15, 16). Chest compressions can be performed on all patients with VADs, with the exception of the Total Artificial Heart (TAH) (SynCardia Systems, LLC; Tucson, Arizona). Patients with a TAH have had their ventricle(s) excised at the time of implantation, and therefore do not benefit from chest compressions, defibrillation, pacing or cardioactive medications; the plastic artificial ventricle is incompressible (14, 17, 18). A large study of adult LVAD patients with cardiac arrest between 2010 and 2018 did show a higher mortality in those who received CPR compared to those who did not. But the authors advise that this finding must be interpreted with caution since the two groups differed based on several variables that were independent risk factors for mortality. Of note, the authors also reported a declining rate of utilization of chest compressions over the time period studied, and this was hypothesized to be due to increasing uncertainty regarding the safety and efficacy of chest compressions in the VAD population (19).

Without the ability to rely on traditional cutoff points such as the loss of a pulse or a drop in heart rate below 60 beats per minute, clinical assessment and judgement of a profound decline in perfusion should serve as the indicator for when to initiate chest compressions in pediatric CF-VAD patients who have an acute circulatory decompensation. Guidelines for CPR, which can be used in all other patients, will not apply in pediatric CF-VAD patients. Thus, even experienced ICU staff who perform CPR regularly have demonstrated uncertainty regarding timing of initiation of chest compressions in CF-VAD patients. In the face of declining continuous variables such as MAP or near infrared spectroscopy (NIRS), clinicians may be uncertain about what cutoff points should trigger chest compressions, or whether to use inotropes alone.

Garg et al. reviewed a series of adult patients with continuous-flow left ventricular assist devices (LVADs) who underwent in-hospital CPR, and compared them to patients without VADs at the same hospital who suffered in-hospital cardiac arrest. They found a statistically significant difference in time to initiation of chest compressions, with the VAD patients experiencing a delay in initiation. They attributed this delay to clinician uncertainty about when to start compressions in this group of patients who are pulseless at baseline. They coined the term “hypotensive electrical activity” (HEA), and created an algorithm to establish cut-off points beyond which chest compressions should be initiated (8). Yuzefpolskaya et al. also published a proposed algorithm for CPR in adult CF-VAD patients, after presenting a case report that demonstrated delayed initiation of chest compressions in a hospitalized adult LVAD patient (9).

Similar to these adult cases, our pediatric cases involved clinician uncertainty about timing of initiation of chest compressions, leading to short delays in resuscitation. Our cases were from a pediatric cardiac intensive care unit at a single large tertiary center that provides a broad range of advanced cardiac therapies. Our proposed CPR algorithm for pediatric CF-VAD patients is written for hospitalized patients in intensive care units. This algorithm was presented several times before multidisciplinary groups for feedback as well as education.

Categorical decision-points we used in our algorithm include determination of VAD function, determination of presence of a palpable pulse, determination of MAP, and assessment for acute change in mental status or drop in ETCO_2_. The published adult VAD CPR algorithms (8–10) recommend use of Doppler to determine presence of (pulseless) arterial blood flow in emergencies. Of note, those algorithms start with a response to an unresponsive patient. Our algorithm starts with response to a low-flow alarm, and initially addresses VAD function, and we account for those with presumed preserved flow by using hemodynamic and clinical parameters to determine need for compressions. Our patients were all hospitalized in intensive care, and their circulatory emergencies first came to clinician attention due to low-flow alarms. Also in contrast, the AHA guidelines for CPR in patients with mechanical circulatory support targets use by emergency medical personnel in the field (14).

Our cut-offs for drop-off MAPs by weight need to be validated in further studies. Even in non-VAD patients, determination of the blood pressure below which chest compressions are indicated (in the absence of non-perfusing rhythm or bradycardia) remains a controversial topic. This is true even in adult medicine where it is more likely (than in pediatrics) that one blood pressure number chosen may apply to most patients. Harper et al., in an editorial in 2020, proposed a systolic blood pressure <50 mmHg as a trigger to initiate chest compressions in the anesthetized adult patient (20).

Our choice of ETCO_2_ < 20 mmHg also needs to be further studied, but is extrapolated from existing resuscitation literature that associates an ETCO_2_ < 20 mmHg during CPR with poor cardiac output from poor CPR quality (21).

Our algorithm is intentionally simple, and does not include details on how to perform CPR. Users of this algorithm are thus referred to existing guidelines for pediatric basic and advanced life support, once the decision tree has suggested to start chest compressions. Also for simplicity, our algorithm does not provide extensive guidance on how to resuscitate pediatric VAD patients with low-flows who do not require chest compressions.

## Conclusion

There is minimal existing literature demonstrating the scope of the problem of circulatory collapse requiring chest compressions in patients with CF-VADs. Published literature does not include pediatric patients. Our case series of 4 pediatric CF-VAD patients demonstrates the significant morbidity and mortality that can occur. The already published literature as well as our case series demonstrate that there may be delays in resuscitation due to clinician uncertainty about when to begin chest compressions in this population that would have “hemodynamically-stable PEA” at baseline.

A CPR algorithm, such as we have proposed, that guides decision-making may lead to earlier and more appropriate resuscitation in pediatric CF-VAD patients with cardiovascular emergencies. Further investigation is necessary to determine the applicability of our algorithm across different pediatric centers, as well as its use in reducing time to initiation of appropriate chest compressions.

## Data Availability Statement

The datasets presented in this article are not readily available because of ethical/privacy restrictions. Requests to access the datasets should be directed to the corresponding author.

## Ethics Statement

The studies involving human participants were reviewed and approved by UT Southwestern Medical Center IRB. Written informed consent from the participants' legal guardian/next of kin was not required to participate in this study in accordance with the national legislation and the institutional requirements.

## Author Contributions

IE: data collection (case series), data collation, creation of algorithm, and writing of manuscript. PY: editing of algorithm, review of literature, and review of manuscript. Both authors contributed to the article and approved the submitted version.

## Conflict of Interest

The authors declare that the research was conducted in the absence of any commercial or financial relationships that could be construed as a potential conflict of interest.

## Publisher's Note

All claims expressed in this article are solely those of the authors and do not necessarily represent those of their affiliated organizations, or those of the publisher, the editors and the reviewers. Any product that may be evaluated in this article, or claim that may be made by its manufacturer, is not guaranteed or endorsed by the publisher.
