# Supplementary material for: Chest Compressions in Pediatric Patients With Continuous-Flow Ventricular Assist Devices: Case Series and Proposed Algorithm
Source: Front Pediatr. 2022 Jun 21;10:883320. doi: 10.3389/fped.2022.883320 (PMC9253534; doi:10.3389/fped.2022.883320)
Supplement: Supplementary file 1 [file Table_1.pdf]

<u>Supplementary Table S1</u>: Patient- and Device-Related Problems to Consider with Decreased Continuous-Flow LVAD Output

**Device**	**Patient**
• Loss of power<br>• External driver failure<br>• Driveline failure or damage [e.g. HeartMate-3]<br>• Cannula disconnection [e.g. PediMag, CentriMag]<br>• Pump thrombosis	• Right ventricular failure<br>○ Myocardial dysfunction<br>○ Arrhythmia<br>○ Increased pulmonary vascular resistance<br>• Pericardial tamponade<br>• Preload: Hypovolemia<br>• Afterload: Increased systemic vascular resistance (will result in decreased CF-VAD flow)

*This simplified table summarizes key patient- and device-related issues to consider when there is low-flow or evidence of decreased tissue perfusion in a patient with a CF-VAD in the left ventricle position.
*The type of VAD significantly influences how to troubleshoot.
*The problems listed as right ventricular failure can be considered when the patient has two ventricles and a native right ventricle, but will not apply in single ventricles or in the presence of an RVAD.

VAD, ventricular assist device. *LVAD*, left ventricular assist device. *RVAD*, right ventricular assist device. *CF-VAD*, continuous-flow ventricular assist device.
